# Next-generation gluten-free noodles: integration of hydrocolloids, fibers, and bioactive compounds

**DOI:** 10.1007/s10068-025-02081-w

**Published:** 2026-01-12

**Authors:** Oladeji Solomon Oluwole, Farhan Mohd Said, Nur Fathin Shamirah Daud, Fatmawati Adam

**Affiliations:** 1 Faculty of Chemical and Process Engineering Technology, Universiti Malaysia Pahang Al-Sultan Abdullah, Lebuh Persiaran Tun Khalil Yaakob, 26300 Gambang, Pahang Malaysia; 2 Department of Physical Sciences, Landmark University, PMB 1001, Omu-Aran, Kwara State Nigeria; 3 Centre for Research in Advanced Fluid and Processes, Universiti Malaysia Pahang Al-Sultan Abdullah, Kuantan, Pahang Malaysia

**Keywords:** Gluten-free noodles, Nutritional quality, Hydrocolloids, Bioactive compounds, Consumer acceptance

## Abstract

Growing consumer interest in gluten-free food, fueled by celiac disease, gluten intolerance, and health-oriented consumers, has prompted the investigation into gluten-free noodles (G-FN). However, the absence of gluten poses a serious challenge to formulating noodles with desirable texture and nutritional acceptability. This review critically evaluates sustainable and functional solutions for enhancing G-FN formulations, focusing on hydrocolloids, dietary fibers, and bioactive compounds. Recent ingredient blends such as hydrocolloids mimicking gluten network, fibers managing bio-accessibility of starch and hydration, and bioactive compounds improving the nutritional value are highlighted in enhancing product performance and stability. Industrial implications are considered for enhanced dough processing, shelf-life, and nutrition labelling compliance. Sensory quality and consumer acceptance are discussed along with industrially relevant solutions. Future research directions are proposed to guide the nutritionally fortified formulation and universally accepted G-FN by health-oriented consumers, and to offer useful guidance for next-generation G-FN that aligns with consumer and regulatory demands.

**Supplementary Information:**

The online version contains supplementary material available at 10.1007/s10068-025-02081-w.

## Introduction

In many countries, noodles form a major part of daily nutrition and are produced from rice, corn, wheat, and other grains and starches, including pulses, potatoes, and sweet potatoes (Liao et al., 2021a, b; Obadi et al., 2020; Oladeji et al., 2025). According to reports, 40% of the wheat consumed in Asia is linked to noodles, accounting for about 12% of global wheat (Obadi et al., 2022). However, the rising occurrence of gluten health conditions including wheat allergy and celiac disease has propelled the demand for gluten-free noodles (G-FN). Wheat noodles are restricted for consumers with cardiovascular disease and diabetes due to high glucose in the blood (Liu et al., 2018). The demand for gluten-free foods increased to over USD 7.91 billion in 2024 and is projected to reach USD 14.67 billion by 2032 (Semplicini, 2021). Nevertheless, creating G-FN involves some difficulties, especially regarding texture, flavor, and nutritional quality such as vitamins, fibers, and minerals (Guardianelli et al., 2019). The growing interest in G-FN has propelled extensive research by food scientists on its preparation from pseudo-grains and fortifications with additives (Drub et al., 2021).

In conventional noodles, gluten creates an uninterrupted protein network that offers structural strength, elasticity, and chewiness. The absence of gluten in G-FN produces foods that are brittle, sticky, or prone to disintegrating during cooking (Obadi et al., 2022). However, incorporating modified starches, native starches, and hydrocolloids (HCs) is considered a substitute for gluten or wheat flour in G-FN (Han et al., 2020). For instance, the tensile properties of G-FN produced from rice/rice starch and xanthan gum (XG) are lower than wheat noodles (Raungrusmee et al., 2020). In a similar study, pregelatinized flour/starch was reported to provide a viscous characteristic and trap air bubbles in G-FN (Cheng et al., 2020; De Arcangelis et al., 2020). The modified starch enhances the dough quality and texture, besides improving the water and air holding capacity (Han et al., 2021; Miyazaki et al., 2006). The nutritional content of G-FN has been improved with the addition of legume flours, pseudocereals, and dietary fibers (Kraithong et al., 2023). Locust bean gum, XG, and oat fiber are reported to enhance the flavor and textural attributes of G-FN (Inglett et al., 2005; Raungrusmee et al., 2020). In addition to the structural and nutritional enhancements, these additives could significantly prevent some diseases including diabetes, obesity, colon cancer, and coronary heart disease (Martín-Esparza et al., 2018). As a result, G-FN has gained immense interest in human nutrition. Also, factors such as protein structuring and starch blends have been reported to improve the sensory and textural attributes of G-FN (Martín-Esparza et al., 2018). These innovations seek not only to mimic the appealing characteristics of wheat noodles but also to structurally and nutritionally develop products that appeal to health-focused consumers.

In recent years, functional additives such as HCs, dietary fibers, proteins, starch mixtures, and bioactive compounds have significantly improved both nutritional content and structural quality of GF-N. Most of these reports were based on the formulation techniques; however, their mechanistic contributions during processing are not extensively documented, especially in starch retrogradation and its water–macromolecule interaction modulation (Heo et al., 2013). Given that retrogradation influences texture and sensory properties, an understanding of how functional ingredients impact these molecular realignments is necessary. Taking a step beyond ingredient function cataloging, this review synthesizes evidence on compositional and nutritional effects, namely, how HCs, fibers, and bioactive compounds modify macronutrient balance, dietary fiber supplementation, and bioactive compound retention or supplementation in G-FN. Synthesizing mechanistic information with formulation effects, we outline a conceptual model for the design of G-FN products that meet both technological and nutritional fortification objectives, amid increasing consumer demand spurred by gluten intolerance and health-oriented diet choices.

## Gluten-free noodles and the drawbacks

**Fig. 1:**
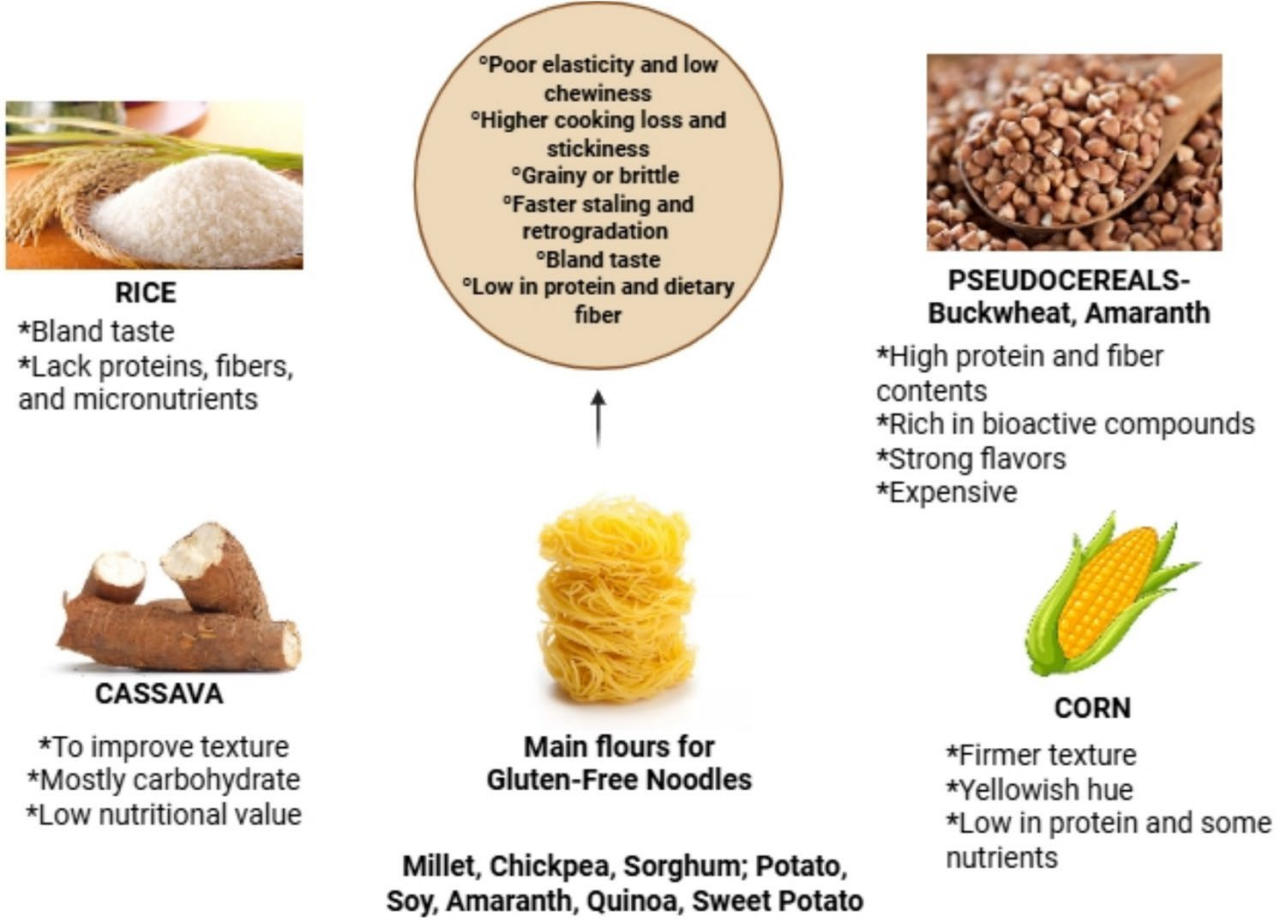
Main flours used in G-FN- Advantages and limitations (Adopted from Kaur et al., 2022 and Woomer and Adedeji, 2021)

G-FNs are globally regarded as an alternative food for gluten-intolerant and diabetic patients because of the absence of gluten (Liu et al., 2018). G-FNs are traditionally made using gluten-free flours derived from cereals, pseudocereals, legumes, and tubers. The most frequently used base flours are sorghum, millet, corn, rice, quinoa, amaranth, buckwheat, chickpea, and cassava (Fig. 1) (Woomer and Adedeji, 2021). They are selected primarily for the absence of proteins involved in gluten formation and for their availability and affordability across different regions globally. Among these, rice flour is the most widely used as it is neutral in flavor, hypoallergenic, and white, being closest to that of normal wheat noodles. However, rice flour lacks protein, fiber, and micronutrients, which can be substituted with nutrient-dense substitutes such as pseudocereals, legumes, or dietary fibers such as psyllium or inulin (Kaur et al., 2022). Corn flour contributes a coarser texture but lacks protein content and contributes a yellowish hue (Kaur et al., 2022). Gluten-free alternative grains such as buckwheat, amaranth, and quinoa offer more nutrient-rich profiles with enhanced flavours; however, they are cost-prohibitive, thus affecting their applications in G-FN (Kaur et al., 2022).

Traditional wheat noodles are composed of gluten (fortified with vitamins and minerals), which is responsible for their elasticity and stability when cooked (Naqash et al., 2017). However, the G-FN poses several complications in terms of cohesion, structure, and elasticity, leading to undesirable physical or structural characteristics, such as brittleness with lower cooking and sensory qualities. Gluten enhances the viscoelastic characteristics of wheat dough, offering elasticity, extensibility, and cohesion; hence, its absence increases fragility and brittleness in G-FN (Naqash et al., 2017). The lack of gluten proteins is a stumbling block to achieving desirable sensory attributes, hence leading to decreased cooking consistency, high starch loss, undesirable firmness, and taste (Matos and Rosell, 2012; Naqash et al., 2017). Gluten-free starches and flours are generally low in protein, fiber, and micronutrients, leading to nutritionally inferior products. The enhancement of their nutritional quality involves partial substitution with nutrient-rich pseudocereals (buckwheat, quinoa), fibers (inulin or β-glucans, and resistant starch), antioxidant-rich powders (green tea, spinach, beetroot), and legume flours (lentil, chickpea). The fortification with iron, calcium, zinc, and B-complex vitamins can mitigate the nutrient deficiency without altering sensory or functional properties (Pellegrini and Agostoni, 2015).

### Functional ingredients in gluten-free noodles

Innovations in G-FN are reshaping the realm of gluten-free diets and overcoming persistent issues related to texture, nutrition, and stability. Through ingredient modifications, researchers are developing products that match the quality of conventional wheat noodles while providing improved nutritional advantages. G-FN is brittle, fragile, with reduced elasticity; hence, overcoming these challenges using ingredient modifications is a critical area of innovation. The effects of ingredients such as HCs, fibers, and proteins on the texture, sensory, rheology, and nutritional features of G-FN are extensively discussed.

### Hydrocolloids: structure and compositional impacts

HCs, including guar gum (GG), carob gum, carrageenan, sodium alginate, arabic gum, konjac glucomannan, and XG, comprise a diverse collection of long-chain polymers primarily recognised as polysaccharides, which have been extensively reported in the production of G-FN (Yemenicioğlu et al., 2019). HCs are thickeners or viscosity-increasing agents capable of altering the textural and rheological attributes of G-FN (Liao et al., 2021a, b). These polysaccharides exhibit various traits, such as linear and branched chain formats, along with positive, negative, and neutral charges, and molecular conformations in solutions, besides gelling and thickening properties, which enhance their functional diversity, improve water retention, and reduce noodle cooking loss (CL) (Table 1) (Kraithong et al., 2023). Several HCs have augmented the sensory, rheology, shelf-life, and stability of G-FN, hence replicating the quality of wheat noodles (Huang et al., 2022; Liang et al., 2022; Liu et al., 2022). The impact of XG and GG on the rheology, microstructure, physicochemical characteristics, and quality of G-FN will be extensively studied.

**Table 1:** Commonly used HCS in G-FN

Hydrocolloids	Source(s)	Properties	Applications	Limitations	References
Konjac glucomannan	*Amorphophallus* konjac (tubers)	High molecular weight with high gel-forming properties	Applicable to noodles with low calories	Odour, brittle texture, and pH sensitive	Tan et al. (2019)
Xanthan gum	*Xanthomonas campestris* (fermented)	Pseudoplastic, stable over a high range of pH and temperature	Strengthen the gluten-free dough and shelf life	Restricted gelling leading to a sticky mouthfeel	Nsengiyumva and Alexandridis (2022)
Methylcellulose	Cellulose (chemically modified)	Thermogelation, water retention	Improves the chewiness and elasticity of G-FN	Synthetic origin, controlled in some markets	Saiki et al. (2022)
Guar-gum	*Cyamopsis tetragonoloba* (seed endosperm)	Hydrophilic in cold water and high viscosity	Improve dough water retention, elasticity, and viscosity	A high amount could cause firmness	Krstonošić et al. (2021)
Gum Arabic	*Acacia* trees (exudates)	Stabiliser and emulsifier	Decreases stickiness and improves mouthfeel	Low viscosity, reduced effect on texture	Musa et al. (2019)
Pectin	Apple pomace and citrus peel	Anionic gel-forming polysaccharide	Provides softness and water holding in noodle-fortified fibers	pH sensitive and divalent cation-sensitive	Rolandelli et al. (2024)
Sodium alginate	*Laminaria* and *Macrocystis* (Brown seaweeds)	Gel formation in the presence of calcium ions	Enhanced firmness and heat stability	Sensitivity to ionic concentration becomes brittle	Jiang et al. (2025)

GG and XG are extensively used in G-FN due to their higher water-holding, thickening, and stabilising abilities. GG enhances dough cohesion and viscosity through the formation of a hydration shell with a similar structure to gluten, while XG strengthens elasticity, cooking stability, and texture through its shear-thinning and heat-stable properties. Gums synergistically interact with starches and proteins, reduce CL, and improve mouthfeel. Their gums are generally recognised as safe (GRAS) and compatible with a large portion of gluten-free flours, and their clean-label appeal sets them up for bolstering the structure and taste features of G-FN.

### Xanthan gum

**Fig. 2:**
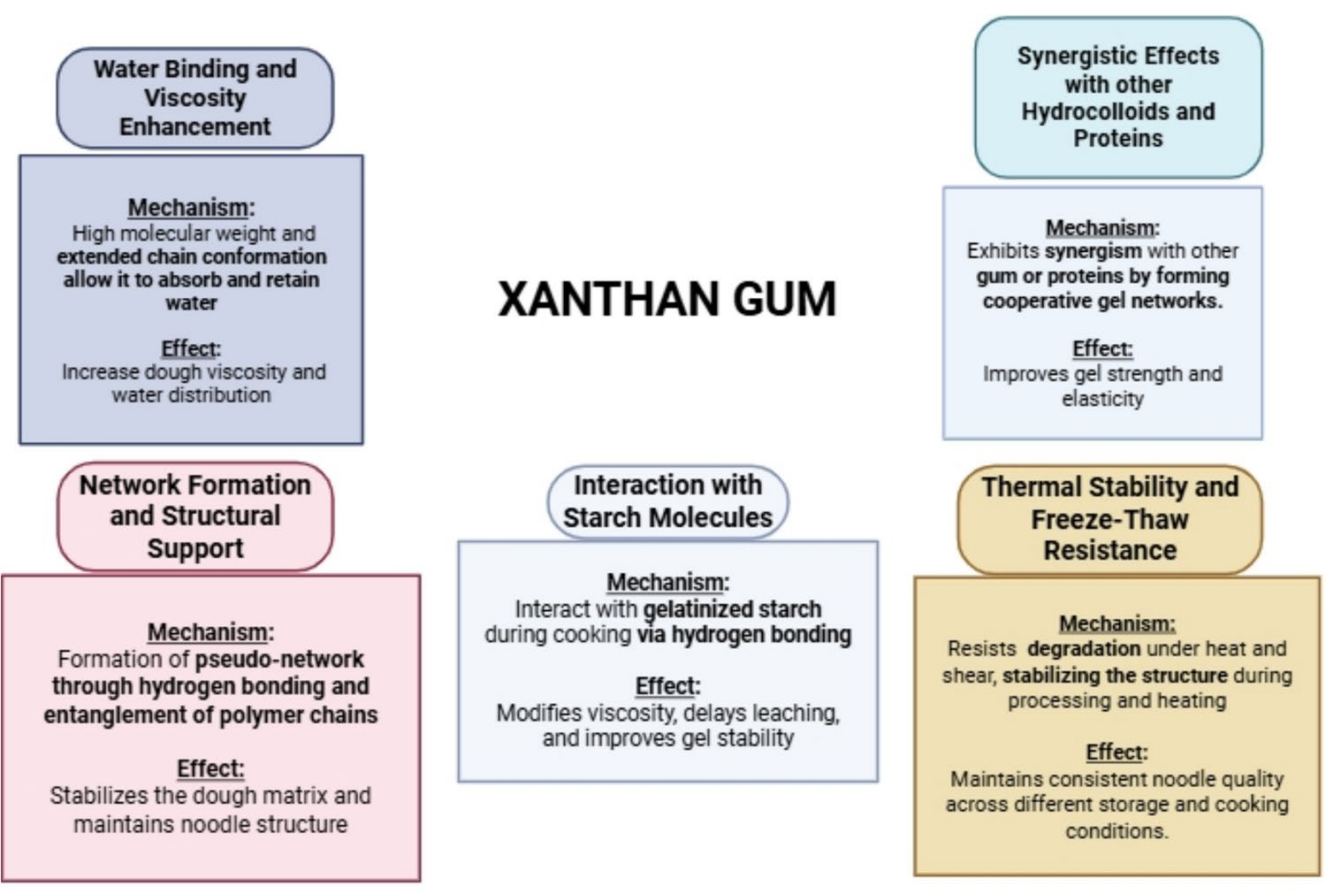
Mechanism of action of XG in G-FN (modified from Carmona et al., 2015; Kumar et al., 2024; Liu and Yao, 2015)

XG is extensively used in noodle production, and this could be due to its bio-adhesive, biodegradable (cellulose-like backbone structure), biocompatibility, and non-toxic properties, which were certified by the US FDA in 1969 (Luo and Wang, 2014). They are naturally occurring, hydrophilic, neutral, and water-soluble polysaccharides, well-organized in conformation, and highly stable in alkaline and acidic media because of their complex structure (Kumar et al., 2024). At low ionic strength or high temperatures, they exhibit a disordered conformation and a helix structural conformation (single or double) at low temperatures (Liu and Yao, 2015). In an aqueous solution, XG acquires a coordinated viscosity (at low concentrations), as the –OH (hydroxy) and –COOH (carboxyl) polar groups to form intramolecular and intermolecular hydrogen bonds, contributing to pseudo-plastic behavior attributed to their hydrogen bonding interactions and high molecular mass (Carmona et al., 2015). Their potential to restore viscosity (pseudo-plastic nature) under shear gives the product excellent pourability (Fig. 2) (Robless et al., 2004).

In G-FN, XG functions as a viscosity enhancer via physical interactions and hydrogen bonding with starch and non-gluten molecules. These interactions improve water binding and thickening ability, dough rheology, and quality of G-FN (Kumar et al., 2024). The addition of XG has been widely reported to enhance the rheology, sensory, and structural development of G-FN (Table 2). The roles of XG as viscosity and sensory enhancers were explored in G-FN produced from Pathumthani 80 rice (RD 31- a non-glutinous, high-amylose aromatic rice cultivar) and native autoclaved resistant starch (NARS). As XG increased from 0.625 to 5%, the resistant starch (RS) content decreased from 44.06 to 18.40. As XG increased, inhibition of starch retrogradation also increased, interfering with connections or linkages in the amylose chain, hence leading to a reduction in RS formation. Besides, the glycemic index (GI) also reduced from 61.15 to 50.15 (Raungrusmee et al., 2020). This trend was analogous to the results of Srikaeo et al. (2018) and Milde et al. (2022), where the rate of GI and starch digestion of dried fermented rice and cassava noodles reduced as more XG was added. This could be linked to the effect of HCs on the microstructure, pattern of hydration, and starch of noodles (Srikaeo et al., 2018). The incorporation of XG (2.5%) in the G-FN induced porosity and hollow structure into the microstructure of defatted rice bran. This porosity was not observed in G-FN produced with resistant rice starch alone (Raungrusmee et al., 2020). This was in agreement with the findings of Javaid et al., where the addition of XG caused porosity in the microstructure of potato instant noodles. The induced porosity is responsible for the observed CL in the G-FN (Javaid et al., 2018). Moreover, hydrogen bonding in polysaccharide gum (between inner and exterior chains) and starch mixture forms a stable and rigid gluten-like network and helps to improve dough texture, structure, viscosity, and dietary fiber content (Padalino et al., 2016). The increase in water absorption capacity (WAC) of the rice noodles is linked to the presence of the OH group, which forms hydrogen bonding with the water molecules and starch in the G-FN (Raungrusmee et al., 2020).

**Table 2:** Effects of XG on the properties of G-FN

Base flour used (XG)	Observed effect on noodles	Sensory evaluation	Cooking quality	Effects/Implications	References
Defatted rice bran (Pathumthani 80 rice) (0.625, 1.25, 2.5, and 5.00%)	As XG increases: RS ↓ (47.22–18.40) TS ↓ (92.82–60.41) GI ↑ (50.15–61.15) TS ↓ (92.82–60.41)	AR ↑ (6.23–7.06) CL ↑ (6.07–6.92) FL ↑ (5.54–6.62) SFt ↑ (5.23–6.77) STs ↑ (5.15–6.54)	CT ↑ (11.50—14.00) CL ↓ (3.96—2.19) WA ↑ (%) (12.00—63.00)	Improved texture and sensory acceptability, but higher GI suggests limited nutritional benefit	Raungrusmee et al. (2020)
Maize (0.2, 0.6, and 1.0%)	As XG increases: PV ↑ (1193–1213) TV ↑ (1014–1074) BD ↓ (181–121)	HD ↑ (2621–3541) CO ↓ (0.32–0.185) SP ↑ (0.148–0.256) CH↑ (48.25–91.54)	CL ↓ (4.71–2.84) WA ↑ (%) (82.86–57.36)	Enhanced cooking stability and elasticity, with higher chewiness improving consumer appeal	Jiang et al. (2025)
Fermented rice (0.05 and 0.10 g/100 g wet basis)	As XG increases: GI ↑ (103.84–105.80) HI ↑ (91.73–93.39) RS ↓ (1.29–1.17) NRS ↓ (72.31–72.07) TS ↓ (73.60–73.23)	FR ↑ (43.63–45.05)	CL ↑ (0.93–1.13) WA ↑ (198.88–210.83)	Improved firmness and hydration but increased GI reduces suitability for low-GI diets	Srikaeo et al. (2018)
Rice (2.5 and 5 g/ kg, flour basis)	As XG increases: RS ↑ (14.46–16.13) RDS ↑ (67.21–64.65) GI ↓ (85.02–83.64) SDS ↑ (18.10–19.22) RC ↓ (12.45–12.14)	HD ↓ (498–492) CO ↑ (0.51–0.54) TF ↑ (15–23) CH↑ (248.20–262.45)	NA	Balanced nutritional profile with reduced GI and improved digestibility; moderate hardness may affect consumer preference	Huang et al. (2022)

↓ Decrease, ↑ Increase

*TS* tensile strength (N)^ns^, *CT* cooking time (mins), *CL* cooking loss (%), *WA* water absorption (%), *Cn* concentration, *AP* appearance (^ns^); *CL* color (^ns^), *FL* flavor (^ns^), *SFt* softness (^ns^); *STs* stickiness (^ns^), *HD* hardness (N), *CO* cohesiveness (no unit), *SP* springiness (mm), *CH* chewiness (mJ), *BD* breakdown, *PV* peak viscosity, *TV* trough viscosity, *HI* hydrolysis index (%), *NRS* non-resistant starch (%), *FR* firmness (N), *RC* relative crystallinity, *TF* total flavor, *SDS* slowly digestible starch (g/100 g), *NA* not available

XG is an anionic HCs that forms stable electrostatic interactions with positively charged protein molecules, forming improved viscous and elastic characteristics (Zhang et al., 2023). They act as a structure-forming agent that enhances viscosity by forming complex gel networks in aqueous medium (Leyva-Porras et al., 2017). According to Jiang et al. (2025), the incorporation of XG (0.2, 0.6, and 1.0%) into maize composite dough significantly increased the viscosity, elasticity, and quality of composite dough by improving the loss modulus (G′′) and storage modulus (G′). The elasticity observed by XG-fortified composites and trends observed in G’ and G′′ are due to the formation of a weak gel at low shear rates and strong hydration capacity (Xu et al., 2022). In comparison with sodium carboxymethyl cellulose (CMC–Na) and sodium alginate, XG has minimal effect on the loss factor of the composite flour dough, while CMC-Na and sodium alginate increased the loss factor, resulting in an increased viscosity. This trend is due to CMC-Na and SA establishing robust electrostatic interactions with protein molecules, therefore enhancing the gluten network (Jiang et al., 2025; Lazaridou et al., 2007).

Several reports have shown that XG enhances the viscosity and texture of G-FN, and these are attributed to their mechanisms of action. In an aqueous solution, the mechanism of action of XG involves the hydration and expansion of multifaceted helical chains forming a weakly entangled network that absorbs water molecules; hence, a viscous, stable matrix is formed. The matrix formed exhibits shear-thinning attributes which are stable against pH and temperature alterations and with other HCs to strengthen the gel network and improve the texture (Jiang et al., 2025). The addition of XG, CMC-Na, and sodium alginate increased the creep strain, showing a decrease in the dough’s resistance to degradation. These trends increased as more CMC-Na and sodium alginate were added; however, a slight decline was observed in XG-induced dough, indicating XG’s ability to reduce hardness, increase elasticity, and recover dough (Onyango et al., 2009). Another possible factor is the adhesive nature of XG interacting with starch particles and macromolecules such as protein, resulting in a gluten-like network, resulting in a significant reduction in the pores, while enhancing the density of the dough (Jiang et al., 2025).

The starch adhesion of gluten-free flours could be enhanced by incorporating XG, which increases the absorption of water and permits gelatinization to occur at a lower temperature (Kaur et al., 2015). The interaction between XG and amylose (crystal zone) of starch particles increases bonding with water and improves the viscosity, hence increasing the peak viscosity (PV), breakdown (BD), and pasting temperature (PT) (Bi et al., 2022). Adding XG could decrease the rate of starch hydrolysis and increase the slowly digestible starch (SDS) content of rice noodles. XG-complexed rice noodles had a greater extent of hydrolysis and no observed changes in RS content when compared to rice noodles (Srikaeo et al., 2018). A higher concentration of XG improved the hydration qualities of rice flour, which intensified the penetrability of enzymes into starch molecules (Raungrusmee et al., 2020).

### Guar gum (GG)

**Fig. 3:**
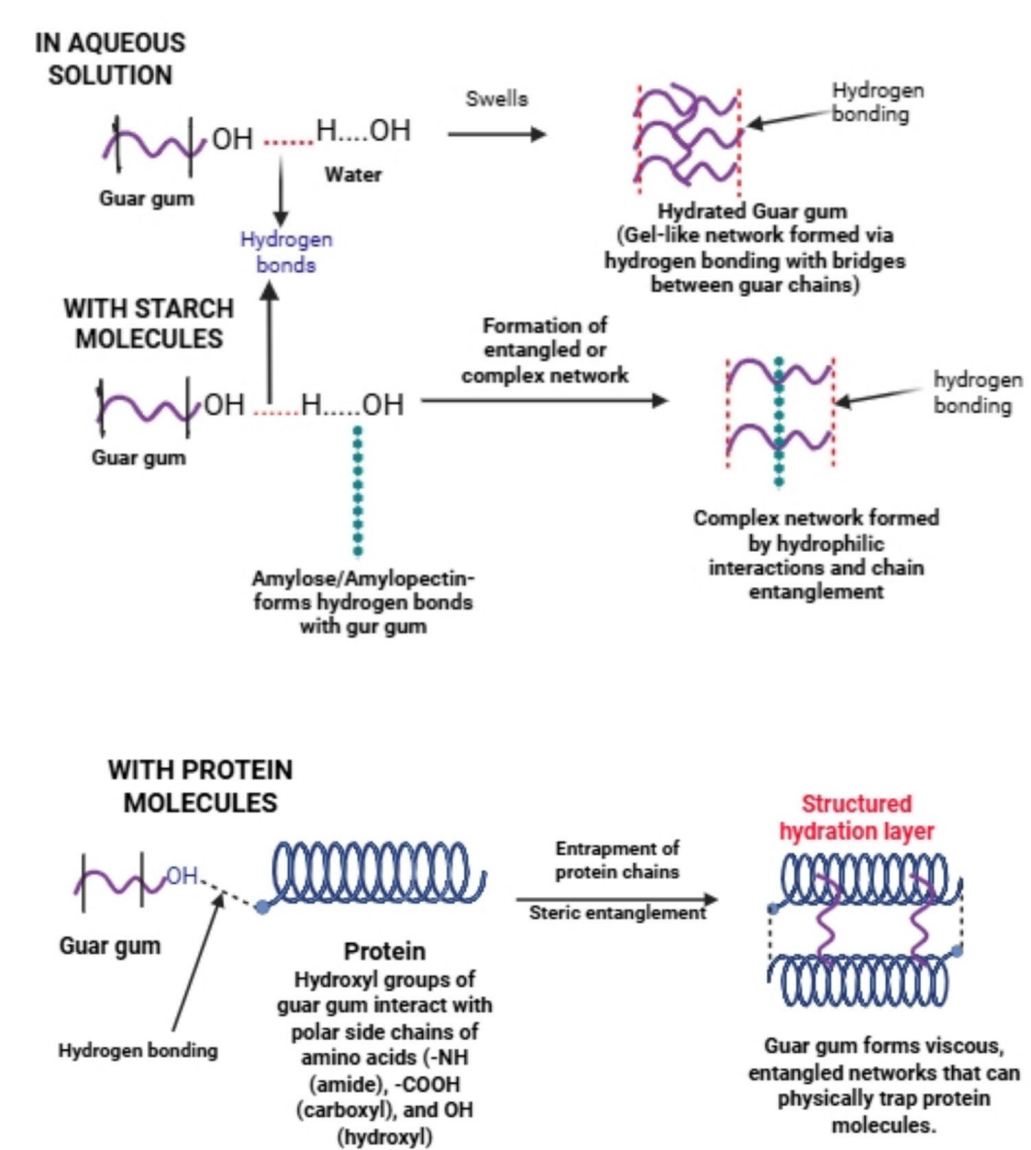
Guar gum mechanism in aqueous, starch, and protein systems [Guar gum acts through extensive hydrogen bonding and water entrapment to yield high viscosity under aqueous conditions. In starch systems, it regulates gelatinization, granule swelling, and slower retrogradation by creating a continuous hydrophilic network. Under protein systems, it attains dispersion stabilization through competitive hydrations and steric hindrances, decreased aggregation, and changed gel strength, elasticity, and water-holding capacity] (Adopted from Rolandelli et al., 2024)

GG is widely used in G-FN due to its ability to trap water and interact with starch molecules. GG develops a hydration shell that supports starch and protein molecules before creating a gluten-like pseudo-network. The interconnected network of starch and proteins formed with GG increases the viscosity, enhancing elasticity and reducing CLs (Fig. 3). Given this, they are commonly used in GF formulations for hardening and achieving predetermined organoleptic and nutritional attributes. The spatial arrangement of the functional groups of these hydrophilic molecules determines their net charge, nature, and strength of interactions with starch and water. Because of these characteristics, they are intriguing materials to enhance the sensory and functional properties of G-FN.

The interactions between the GF starch matrix, GG, and water molecules are significant in the quality of G-FN produced. During gelatinization, starch molecules’ crystallites destabilize, as they lose their molecular order and solubilize. However, the addition of GG has reportedly formed a viscous gel-like structure that is creating a bridge between starch granules, resulting in increased water retention and network density (Rolandelli et al., 2024).

According to Rolandelli et al. (2024), GG (0.5 and 1%) significantly increased the peak viscosity (72.2–71.7ºC), hot paste viscosity (1294–1408 cP), breakdown viscosity (977–894 cP), and final viscosity (1664–1709 cP) of sweet potato-based noodles. The substitution of starch with GG resulted in an increase in all viscosity parameters except for hot paste viscosity. The trends could be linked to the hydrophilic substances competing for water access, which reduced starch’s capability to hydrate and swell. The binding of GG to starch or water molecules strengthened their intermolecular forces, leading to reduced interactions between starch and water, hence lowering gel viscosity (Rolandelli et al., 2024). According to Lee et al. (2002), GG reduced the retrogradation rate because of the reduction in amylose leaching and water-starch linkages. The hydrophilic compounds (GG) restricted starch molecular mobility and amylose and amylopectin linkages through van der Waals and hydrogen forces (Lee et al., 2002; Rolandelli et al., 2024). The incorporation of GG (0.1, 0.3, and 0.5%) on the cooking qualities and physicochemical attributes of fermented hollow dried noodles indicated that GG significantly increased the CL (5.81–5.92%), water absorptivity (178.17–194.70%), expansion index (190.40–191.69%), breaking strength (62.20–66.65 g), and flexibility (7.11–7.13 mm), while splitting rate decreased (25.00–20.00%) as more GG added (Hu et al., 2024). GG enhanced dough texture by making it stronger and more flexible so the gluten-like network can better encapsulate starch granules, which reduced noodle CL (Porwal et al., 2014). GG (0.1%) reduced WAC as it forms gels to restrict starch hydration processes. At elevated concentrations, the WAC increased because of the strong water-binding properties (Hu et al., 2024). The incorporation of GG elevated the bulk strength, and the trend could be linked to the differing thickness of noodle materials and pore formations. According to Hu et al. (2024), GG increased the flexibility of the noodles in a dosage-dependent way so that their dough could become elastic and tougher (Hu et al., 2024).

As a researcher, exploring the incorporation of HCs such as GG and XG offers promising solutions to the current challenges encountered by G-FN, paving the way to more sustainable, quality, and consumer-friendly products.

### Dietary fibers in gluten-free noodles- structure and compositional impacts

In G-FN, fibers with high WAC or the ability to retain moisture within the dough, including cellulose, oat fiber, psyllium, and inulin, are extensively used to improve moisture retention, reduce CL, and enhance cohesiveness (Fig. S1) (Ronda et al., 2013; Wang et al., 2022). Dietary fibers create an essential scaffold in gluten-free dough, maintaining the structure through a pseudo-structural formation process. Insoluble wheat bran and microcrystalline cellulose promote mechanical reinforcement in food matrices and soluble fibers like GG and inulin, which improve viscosity and gel development (Lazaridou et al., 2007; Zhang et al., 2024). Dietary fibers non-covalently interact through hydrogen bonding and electrostatic interactions with gluten-free formulation, plant proteins, and hydrocolloids (Fig. S2). These interactions enhance matrix stability (structural coherence and integrity of the dough network) and suppress phase separation (physical demixing or segregation of dough components such as water, starch, or proteins) during cooking. Increasing matrix stability and reducing phase separation result in improved texture, cooking tolerance, and quality of G-FN (Zhang et al., 2019).

The interaction between dietary fibers with starch and protein molecules in G-FN through water binding and the formation of hydrogen bonds leads to robust network development. Fibers compete with water for absorption, which impacts how starch gels form and how proteins hydrate. The addition of starch allows fibers to slow down gelatinization while increasing viscosity and minimizing retrogradation through its interference with amylose re-association. By bonding to proteins, dietary fibers create weak bonds that contribute to dough stability through their formation of a composite network. A synergetic effect occurs when these interactions create a better texture because they reinforce dough firmness and elasticity while preserving moisture content to compensate for the absence of gluten (Zhang et al., 2019).

### Inulin

Inulin, a naturally occurring fructan consisting mainly of β-(2 → 1)-linked fructose units, is a soluble dietary fiber that exhibits superior multifunctional performance for gluten-free applications. In noodle uses, inulin combines synergistic effects with XG and other hydrocolloids to assist in the formation of a pseudo-gluten network through intermolecular viscosity enhancement and enhance water-binding capacity. Such functions translate to enhanced cohesiveness of the dough, enhanced cook stability, and texture characteristics such as enhanced firmness and elasticity (Padalino et al., 2016). According to Raungrusmee et al. (2020), the addition of inulin (5% w/w) to RD-NARS G-FN containing XG (2.5%) significantly increased the RS content (24.90 to 29.19%) and reduced the GI values (52.7 to 49.20%). These trends could be linked to the ability of inulin to resist enzymatic digestion and cause a reduction in starch digestibility by entrapping starch in a semi-solid gel medium, resulting in low availability of water molecules for gelatinization (Brasil et al., 2011; Delgado and Bañón, 2018). Also, an increase in noodle firmness (0.08 to 0.78 N) while a reduction in the tensile strength (0.39 to 0.24 N) and elasticity (21.0 to 16.49 mm) was observed (p < 0.05). The reduction in elasticity could be linked to the interaction of inulin with starch molecules for hydration, which results in the resistance of starch to swell and the formation of semisolid gel aggregates that alter the structural buildup of the noodle (Mastromatteo et al., 2012).

The effects of inulin have been extensively reported to improve the nutritional value and cooking quality of G-FN (Fig. S3). According to Raungrusmee et al. (2020), inulin (5% w/w) significantly increased the cooking time (13.00 to 27.5 min) and water absorption (62.00 to 79.85%), while a reduction in CL (2.67 to 2.24%) was observed in RD-NARS G-FN (Raungrusmee et al., 2020). The trends in WAC of noodles with inulin could be ascribed to the –OH group in inulin, which forms hydrogen bonding (Afshinpajouh et al., 2014). Nevertheless, inulin has some limitations. At elevated levels of incorporation, excessive water retention is realized, leading to undesirable softness and tackiness of the final product. Through the sheer concentration of fructose, the level of processing heat can propel the Maillard reaction, and unwanted color and flavor shift of the noodle can be induced. Moreover, the limited extensibility prevents inulin from imitating the viscoelastic nature of gluten. The absence of viscoelasticity causes brittle textures (decreases elasticity and tensile strength), prolonged cooking time, excessive firmness, and dry mouth feel (Afshinpajouh et al., 2014; Delgado and Bañón, 2018). To overcome these, inulin can be supplemented with other hydrocolloids or protein-based structuring agents during formulation and production.

### Psyllium

Psyllium (PSY), a naturally occurring polysaccharide and soluble dietary fiber, is extensively used in G-FN due to its viscoelastic, WAC, and gelling properties (Noguerol et al., 2022). According to Fradinho et al. (2020), the cooking properties, nutritional composition, texture, and oxidase resistance of gluten-free foods have been improved with the application of psyllium husk powder (PHP). It has been observed to increase the thickness of the chyme blend in the gut and decrease the rate of digestion of starch by amylase, resulting in a great reduction in the glucose released (Dikeman and Fahey, 2006). The influence of PHP and psyllium powder (PP) on the quality and digestibility of rice noodles (RN) showed that 5% conventional white rice noodles (W-RN), RN made from 5% PHP and 95% DA-RF (5PHP-RN), and RN made from 5% PHP, 2% PP, and 93% DA-RF (5PHP-2PP-RN) were less digested. On addition of 5% PHP and PP, the hardness, adhesiveness, resilience, springiness, and chewiness of W-RN, 5PHP-RN, and 5PHP-2PP-RN increased while PHP and PP reduced (Gong et al., 2024). The noodle hardness and texture are attributed to the high amylose content of DA-RF and the synergistic effects of PHP and PP on WAC and gel formation (Gong et al., 2024; Noguerol et al., 2022). The synergistic effects of PHP and PP enhanced water retention, whereas decreased breakage rates, turbidity, and CL observed in noodles negatively affected their quality (Fradinho et al., 2020; Noguerol et al., 2022). The application of PSY to improve G-FN faces some limitations because at high concentrations, it absorbs too much water, resulting in pasty textures, negatively affects starch gelatinization and firmness, and imparts an earthy taste which can disturb noodle consistency when processed with other ingredients. However, regulated PSY additions could positively affect the texture of G-FN due to their dietary fiber content (Noguerol et al., 2022). Incorporating PSY improves noodle texture and cooking quality while reducing starch digestibility (Table 3).

**Table 3:** Effects of psyllium on noodle quality and digestibility

Based flour and psyllium	Texture profile	Noodle quality	Digestive properties	Implications/Effects	Reference
5PHP-RN	Hardness: 1015.46 g Adhesiveness: -173.20 g Springiness: 78.8% Chewiness: 754.52	WA: 15% CL: 0.44%	Total Starch Digestibility: 61.55% Digestibility: Low	The incorporation of PHP and PP produced firmer, less sticky noodles with improved water retention, elasticity balance, and slower starch digestion, resulting in a better glycemic response	Gong et al. (2024)
5PHP-2PP-RN	Hardness: 1054.87 g Adhesiveness: -116.90 g Springiness: 67.94% Chewiness: 821.60	WA: 24% CL: 0.48%	Total Starch Digestibility: 62.48% Digestibility: Low		
50 PG: 50 rice (PHP of 3, 4, and 5%)	Adhesiveness: 0.035–0.055 Ns ↑ Firmness: 1.55–2.75 N ↑	WA: 43–42% CL: 1.4–0.8% ↓	Carbohydrate: Optimized 50 PG:50 Rice = 93.99% Control* = 97.45%	Increasing PHP resulted in higher pasta firmness, decreased carbohydrate digestibility. However, PHP did not affect WA significantly but has decreased the CL	Fradinho et al. (2020)
Soaked-and-dried soybean: Psyllium (95:10, 75:25, 60:40)	Hardness: 1570.52–1834.62 g ↑ Springiness: 0.53–0.43 ↑ Gumminess: 747.81–1071.22 ↑ Cohesiveness: 0.48–0.58 ↑ Chewiness: 397.08–464.29 ↑	WA: 59.47–64.16% ↑	NA	The texture profile and WA increased as the psyllium content increased	Bak et al. (2022)
50 Rice starch: 50 corn starch 3 PHP 1.5 PHP: 1.5 GG 1.5 PHP:1.5 LBG	Firmness: 190 g 200 g 230 g	CL: 11.63% 9.79% 8.23%	NA	Combining PHP with LBG enhanced the cooking quality and firmness of noodles	Aktaş & Erten (2024)
MRF + PHP (PHP of 0.75, 1.5, 2.25, and 3% (wt/wt))	Firmness: 1.64–1.77 N ↑ Hardness: 20.79–21.08 N ↑ Cohesiveness: 0.38–0.38 Springiness: 0.51–0.54 mm ↑ Chewiness: 4.19–4.49 Nmm ↑	WA: 115.67–111.15% ↑ CL: 19.97–17.69% ↑	Reducing sugar release: MRF = 150 mg/g MRF + PH2.25 = 200 mg/g	PHP had reduced WA and CL. However, increased PHP enhanced firmness and improved reducing sugar release, but no significant difference on the texture profile analysis	Raherison et al. (2025)

↓ Decrease, ↑ Increase

*5PHP-RN* (5% PHP + 95% DA-RF), *5PHP-2PP-RN* (5% PHP + 2% PP + 93% DA-RF), *DA-RF* double enzyme combined with annealing treated rice flour, *PHP* psyllium husk powder, *PP* psyllium powder, Control* 100% rice flour, *PG* psyllium gel, *GG* guar gum, *LBG* locust bean gum, *MRF* modified rice flour, *WA* water absorption, *CL* cooking loss, *NA* not available

### Other dietary fibers

Dietary fibers, such as cereal β-glucans, which can reduce insulin levels, blood serum, and blood glucose, as well as low-density lipoprotein cholesterol, have been incorporated into G-FN (Du et al., 2019). These fibers not only boost the health conditions of the consumers but also improve the nutritional composition of G-FN (Ronda et al., 2013). The incorporation of fiber-rich ingredients obtained from natural fibers such as chia seeds, flaxseed, oat bran, and PSY husk into G-FN formulations has boosted their dietary fiber composition. In addition to nutrient fortifications, they enhance the noodle structure, dough elasticity, texture, mouthfeel, and water retention capacity (Brennan, 2005). Additionally, the utilization of vegetable and fruit isolates, such as citrus and banana peels, beetroot, spinach, and carrots, enhances the fiber content, as well as the flavor and color, of G-FN. This has been reported to reduce GI by improving cooking quality and reducing starch digestibility (Dziki et al., 2014). RS obtained from modified corn starch and green bananas has improved the dietary fiber content of G-FN and improved gut health by stimulating the growth of essential gut bacteria (Birt et al., 2013).

The combination of extrusion and cellulase treatment on okara fiber enhanced noodle quality by improving texture, cooking properties, and appearance, although starch digestibility decreased. Noodles containing 4.0% cellulase-treated okara produced similar attributes to wheat noodles, exhibiting a predicted GI below 55. Data showed that okara fiber decreased noodle water movement due to less water competition and strengthened noodle structures in high-fiber formulations (Xie et al., 2025).

### Bioactive compounds and health-promoting properties

Due to the unique characteristic features of G-FN, the nutrients, texture, taste, shelf life, and functionality need to be fortified. Fortifications with bioactive ingredients such as polyphenols, dietary fibers, polysaccharides, amino acids, and natural pigments have significantly enhanced the quality of G-FN (Nie et al., 2023; Zhang et al., 2021a, b). Beyond their culinary demand, bioactive compounds possess significant potential as a natural fortification for G-FN, offering an innovative and health-oriented approach (Chongtham et al., 2021). Their inclusion in G-FN helps improve the chewiness and springiness, masking their odor and acidity, and increasing their flexibility and texture (Song et al., 2024). The functional roles of bioactive compounds in G-FN are extensively discussed in Table 4.

**Table 4:** The roles of bioactive compounds in G-FN

Bioactive compound (level)	Noodle type	Observed effects	Mechanism of action	References
Erythritol (0–40% w/w)	Extruded dried rice noodles	↑ Peak viscosity (4188.43 → 4968.97 cP); ↑ Final viscosity (4176.81 → 4884.92 cP); ↑ Hardness (82.59 → 98.23 g); ↓ Retention time (10.83 → 4.75 min); ↑ CL (12.62 → 29.92%)	Hydrogen bonding between sugar alcohol, starch granules, and water	Gao et al. (2023)
Lysine (1–5% w/w)	Semi-dried brown rice noodles	↓ Hardness (840 → 6349 g); ↓ Adhesiveness (–671 → –792 g·s); ↓ Cohesiveness (0.396 → 0.393); ↓ Chewiness (1916 → 1483); ↓ Gumminess (3355 → 2499)	Strong starch–starch interactions at 5% lysine	Luo et al. (2025)
Cricket powder (5–15%) and Silkworm pupae powder (5–15%)	Rice noodles	↑ Protein (CR: 13.31–24.61%; SP: 12.06–25.85); ↑ Fiber (CR: 1.92–6.36%; SP: 1.57–3.74); ↓ Carbohydrate (CR: 82.70–64.42%; SP: 80.62–55.50); Slight ↑ water activity and moisture content	Starch network disruption, limited swelling, altered water absorption	Li et al. (2025)
*Lactobacillus fermentum* (5–15% v/v lysine fermentation)	Rice noodles	↑ Hardness (4717 → 5275 g); ↑ Resistant starch (11.08 → 12.00%); ↑ Springiness (42.60 → 47.58%); ↑ Gumminess (1471 → 1808); ↑ Chewiness (826 → 860); ↑ PV (1207 → 1417 RVU); ↑ FV (1347 → 1027 RVU); Slight ↓ Onset T (87.63 → 86.50 °C)	Hydrogen bonding of lysine with starch, modifying crystalline structure and strengthening intermolecular forces	Song et al. (2024)
Fructose (incremental addition)	Cassava noodles	↑ Hardness (138.6 → 179.5 g); ↑ Springiness (0.945 → 0.947); ↑ Adhesiveness (149.0 → 76.3 g·s)	Formation and orderly packing of the double helix structure	Yang et al. (2025)

↑ (increase), ↓ (decrease)

*SP* silkworm pupae powder, *CR* cricket powder, *CL* cooking loss

The viscosity, texture, and sensory effects of bioactive compounds used in G-FN could be linked to the chemical interactions between the bioactive compounds, starch granules, and water. This was reported in Erythritol (ERY) fortified rice noodles. According to Gao et al. (2023), the hydrogen bond strength between water and sugar alcohols is greater than the bond strength inside starch granules, which stabilises the granules and minimises swelling during heating (Fig. S4) (Martínez et al., 2015). Bioactive compounds containing sugar alcohols play a dual role when added to starch by binding to starch chains, which protects the granules from breaking apart (Woodbury et al., 2022). The viscosity of final G-FN increases because starch and sugar alcohol phases separate, stopping amylose from leaving the starch matrix but preserving sufficient network connectivity (Van der Sman, 2019). The interactions between Bioactive compounds and water molecules strengthen starch–water bonding and cause a decrease in water mobility while increasing viscosity levels (Martínez et al., 2015).

According to Luo et al. (2025), as the addition of lysine increased (1 and 3%), the texture of semi-brown rice noodles increased; however, at high concentrations (5%), a significant reduction in the parameters was observed (Table 4). The addition of L-lysine demonstrated an improved combination of taste and odour (at 3% addition) because it counterbalances the fermentation-produced acid through buffering action, which became more intense as the lysine concentration increased. The trend observed at 5% lysine could be due to a strong starch network structure at low lysine concentrations (1 and 3%), which strengthens its connections and enhances food texture. High concentrations of lysine led to a starch matrix becoming excessively rigid, causing reduced chewiness while advancing brittleness. Similarly, lysine (3%) exhibited optimal cooking quality with WAC (140.6%), CL (6.1%), and low starch leakage in noodles. Lysine (5%) demonstrated greater breakage (4.6%), lower WAC (129.5%), higher CL (8.4%), and starch leakage (1.2) (Luo et al., 2025). Similarly, the interaction of lysine with starch through hydrogen bonds or alternative chemical bonds transforms the crystal arrangements, leading to increased thermal stability (Yang and Tao, 2008). According to Song et al. (2024), the high onset temperature exhibited by rice noodles after fermentation of *Limosilactobacillus fermentum* and lysine (5, 10, and 15%) could be linked to the increased thermal stability of rice noodles (ΔH) due to the starch hydrolysis that transforms amylopectin in the amorphous region into crystalline regions (Song et al., 2024).

Apart from the sensory and textural properties enhancements, bioactive compounds are reported to improve the nutritional composition in G-FN. According to Li et al. (2025), cricket powder (CP) and silkworm pupae powder (SP) in rice noodles increased the protein content (85% with CP, and 114% with SP). Other nutritional contents are detailed in Table 4. The hardness of the noodles was enhanced with both CP doses, ranging from 10 to 15% because of enhanced protein content and chitin content, while adhesiveness and cohesiveness were reduced, thus improving the texture quality (Park and Kim, 2023). An excessive amount of insect powder negatively affected the noodle structure. The cooking yield and WAC of noodles were reduced by 5–15% insect powder since the swelling of starch and water absorption capabilities being distorted (Kim et al., 2014). According to Yang et al. (2025), the adhesiveness and hardness of cassava noodles were 235.4 and 118.6 g⋅s, respectively. However, the incorporation of fructose (FR) increased the hardness (138.6—179.5 g) with a significant reduction in the adhesiveness of retrograded starch gels from 48.2 to 149.6 g⋅s. The trend could be due to the formation and systematic arrangement of the double helix structure, resulting in a denser, more organised gel structure and increased crystallinity. This contributed to enhanced hardness and a lower adhesiveness of starch noodles after FR incorporation (Yang et al., 2025). Similar findings were reported by Bolger et al. (2021), in which higher starch retrogradation was observed in cupcakes of fructose, which is higher than in sucrose-fortified cupcakes.

## Consumer perception and product attributes affecting G-FN preference

A mix of structural, sensory, and socioeconomic components shapes the acceptance of G-FN. Recent findings uncover how these factors influence consumer choices and how these factors could be enhanced. The factors regarding consumers’ acceptance of G-FN are detailed below:

### Texture and cooking qualities

The acceptance of G-FN by consumers is influenced by its texture and cooking quality. Given the lack of gluten, which plays a key role in elasticity and structural integrity, health-conscious consumers often seek G-FNs that closely resemble traditional wheat noodles (Hatcher et al., 2002). However, this depends on starch and protein contents, HCs, flour mixtures, additives, and processing techniques (Jeong et al., 2017). However, the particle size of starch or flour significantly influences water absorption, starch gelatinization, and surface texture, which determine the cooking and sensory qualities of G-FNs (Jeong et al., 2017; Kim et al., 2019; Pi et al., 2025). The particle size of gluten-free flours significantly affects the texture and cooking qualities through starch damage, water absorption, surface texture, pasting properties, CL, and cooking yield (Jeong et al., 2017; Kim et al., 2019).

### Starch damage, water absorption, and surface texture

Starch damage (SD), water absorption, and surface texture are three major components influencing the texture and cooking qualities of G-FN; however, these determinants depend on the particle size of gluten-free flour. According to Torbica et al. (2012), flours with smaller particle sizes (80–100 µm) subjected to milling and drying processes undergo significant mechanical and thermal pressures, leading to increased SD. In a related study, Sapirstein et al. (2013) reported that the SD increased as particle size reduced; that is, rice flour (140–250 μm) had 3% SD in AACC units (3.62% Farrand units) while 7.96% (in AACC units and 42.03% in Farrand units) was obtained in rice flour 80–100 μm particles. In G-FN, the damaged starch granules of gluten-free flours often exhibit enhanced WAC, leading to a high swelling rate, CL, and soft and spongy texture. These effects reduced the nutritional composition and textural qualities of G-FN due to the leaching of solids during cooking (Kim et al., 2019). Texturally, moderate starch damage helps bind water and helps firmness of the noodle, but too-damaged starch weakens gel structure and leads to very sticky, non-elastic noodles (Sapirstein et al., 2013). Moreover, the extent of starch damage may influence retrogradation behavior, and, in turn, texture and shelf stability during storage. So, the starch damage must be controlled to a great extent for G-FN to exhibit closer similarity to the good features of the traditional wheat noodles. Particle size is closely related to water absorption behavior. Particles with smaller pores have a higher surface area-to-volume ratio, allowing them to become more readily available to interact with water (Jang et al., 2016), which allows for more effective and even hydration. In addition, noodles produced from a small particle size displayed smoother surface characteristics, and consumers prefer this noodle type (Kim et al., 2019). Noodles produced from coarser particles, by contrast, display a rougher surface and more granular texture that can be less appealing to the senses.

### Pasting properties

The pasting behavior is an important criterion for determining the thermal and shear response of the starch when cooked. Higher SD at smaller particle sizes would be reflected as higher peak viscosity, indicating more swelling and gelatinization. Flour with fine particles has low final and breakdown viscosities, which the characteristic of poor gel structures. The effect is attributed primarily to higher surface area, which allows rapid water uptake and restricts water access for complete starch granule swelling. In addition, over-milling can cause disintegration of the starch granules, which lowers gelatinization efficiency and lowers viscoelasticity. The gel matrix formed has poor mechanical strength and lower resistance to heat and shear stress (Kim et al., 2012). Conversely, flours with larger particles exhibit reduced peak viscosities and thus are less capable of forming a cohesive matrix during cooking (Lee and Oh, 2013). Particle size is also critical; a unique morphological property of flour, as well as water interactions, is used in noodle making. This shows that various desirable or good qualities of gluten-based content can be mimicked, but they need to be balanced to enable consumer acceptance.

### Proportions of ingredients

Ingredient composition is accountable for determining the quality, texture, cooking characteristics, and consumer acceptability of G-FN. The type of flour, starch content, and functional additives such as HCs and dietary fibers play significant roles in the structure, sensory properties, and nutritional quality of the noodle, the characteristics that will dictate consumer preference Meenu et al., 2022). G-FN preference by consumers relies substantially on texture, cooking quality, appearance, and health effects perceived (Javaid et al., 2018). Texture and mouthfeel are of the highest importance since consumers want a firm, elastic bite and glossy appearance (characteristics normally offered by gluten). The inability to develop a good gel network or containing excessive bran and low HCs can make noodles fragile, gritty, or soggy with unacceptable resulting sensory impressions (Tian et al., 2024). Additives like PSY, GG, XG, and inulin can enhance or lower cooking quality depending on concentration. Generally, smooth and uniform noodles are preferred over their rough or porous equivalents due to uneven swelling or high bran content (Raungrusmee et al., 2020). However, the ingredients or additives must be balanced with sensory character, as unpalatable taste or texture will override perceived health benefits. Therefore, effective G-FN products must balance structural integrity, sensory acceptability, and nutritional value to fulfill consumer needs and become successful products.

### Shelf life and swelling performance

Packaging and shelf life play a crucial role in preserving the quality, safety, and marketability of G-FN. Moisture level is a principal factor, regulating noodle structure, shelf life, and customer acceptability. Fresh noodles have a short shelf life (7–14 days) due to high moisture content (MC), while dried noodles (10–12% moisture) may be stored for 6–12 months. Packaging protects against the loss of moisture, oxidation, and nutrient deterioration. Starch structure also influences shelf life—amylose-based starches (e.g., corn, rice, cassava) promote noodle stiffness and shelf life, while amylopectin-based starches (e.g., potato, tapioca) yield softer noodles with shorter shelf life (Wang et al., 2024). HCs also regulate water uptake and provide structural integrity (Lazaridou and Biliaderis, 2007). Moisture allows significant starch, protein, and HCs interactions to simulate gluten behavior. Adequate hydration allows starch gelatinization and protein unfolding for improved structure and texture (Ali et al., 2024; Rao et al., 2024). An accurate swelling index, influenced by amylose and resistant starch content (e.g., banana starch), improves cooking quality and avoids disintegration (Adebowale et al., 2012; Hadiyanto et al., 2019). Low moisture causes brittleness due to incomplete interactions between starch and protein, while high moisture produces weak, sticky noodles with low shelf life. Maximizing ingredient interactions and MC is therefore critical for favourable texture, shelf stability, and overall noodle quality (Adebowale et al., 2012).

### Regulatory compliance and labelling

Proper labelling and regulatory compliance of G-FN are important to ensure consumer confidence, safety, and informed choice. For example, regulatory requirements by the Food and Drug Administration and the European Union (No 828/2014) require that G-FN contain less than 20 parts per million of gluten (Maskeliunas and Miyagishima, 2008; Traynor, 2006). These specifications also establish ingredient ratios, such as HCs (0.2–2%), proteins (5–20%), starch (30–70%), and dietary fiber (1–10%) to ensure uniformity and meet consumer needs (Traynor, 2006).

For consumers with gluten intolerance, there has to be proper labelling because trace amounts of gluten could be disastrous to their health. In the UK, emphasis is placed on transparent labelling of ingredients, amounts, and production processes by the Food Standards Agency (Primrose et al., 2010). Consumer feedback, however, indicates errors in incorrect labelling, with 52% of individuals perceiving GF foods as not being properly labelled. Despite this, 89.3% of respondents showed that they identified the “crossed grain” symbol and written declarations on packaging as important (Sielicka-Różyńska et al., 2020). Secondly, front-of-pack nutrition labelling, for example, warning signs for unhealthy nutrients like fat and sugar, is gaining popularity globally. These labelling strategies are proposed to guide healthier food consumption and can play a significant role in influencing consumer food choices (Ares et al., 2023; Crosbie et al., 2023; Song et al., 2024). However, health claims on the label can have a “health halo effect” in which people assume products are healthier due to certain claims, for example, “zero-gluten” or “low fat,” yet the product itself may not necessarily be more nutritious (Andrews et al., 2000; Choi & Reid, 2015). Warning notices can offset this impact, though their effectiveness may be reliant on the type of claim and type of food involved (Centurión et al., 2019).

### Consumer preferences

Consumer demand for G-FN is driven by both sensory attributes and nutritional value, resulting in continuous efforts to improve GF foods (Aguiar et al., 2021; Alencar et al., 2021; Capriles et al., 2023). Studies have revealed that non-gluten consumers are concerned with factors like nutritional information, product ethics, and product information while making decisions (Savarese et al., 2021). Consumers are especially more inclined towards non-genetically modified, non-processed, and organic food (Christoph et al., 2018). Non-gluten foods are also likely to be perceived as healthier, less processed, and lower in calories compared to gluten-containing foods (Prada et al., 2019). Also, “free-from” products are generally perceived by consumers as being healthier in four countries in Europe (Hartmann et al., 2018). In a study involving consumers (ages 7 to 14) with celiac disease, while visual appeal informed product selection, taste had no discernible impact on their choices (Mazzeo et al., 2013). This shows that while appearance is crucial, the sensory perception, particularly flavor could not be as critical in the acceptability of G-FN among children with gluten-related disorders. Accordingly, there is a need for more intensive sensory assessment and product development among children and those with gluten disorders that can meet their requirements and improve their overall experience with the application of gluten-free diets.

## Findings

XG, GG, dietary fibers, and bioactive compounds synergistically enhance the quality, functionality, and nutritional value of G-FN. XG increases viscosity, dough stability, and texture through strong hydrogen bonding with starch and proteins to form a shear-thinning gel matrix that increases water absorption, elasticity, and shelf life and decreases the GI. Similarly, GG enhances cooking quality and textural strength by hydration shell and gluten-like network development, enhancing water-holding and reducing CL. Diets containing fibers such as inulin and PSY enhance the dough matrix strength and resistant starch content, leading to glycemic control and moisture retention, but require balanced inclusion to maintain textural quality. Bioactive compounds like polyphenols, lysine, and insect protein also fortify G-FN through increased protein content, heat resistance, and elasticity by starch-strengthening interaction, providing better functionality and extended shelf life if properly formulated.

G-FN quality relies on the characteristics of main ingredients, such as flour particle size, starch type, HCs, and dietary fibers that affect texture, elasticity, and cooking quality. Small flour particles improve starch damage, water absorption, and swelling, but with gel stability compromised, and need to be well balanced in formulation to provide mouthfeel. Amylose-rich starches and adequate control of moisture improve shelf life and structural integrity. Other than formulation, clean and compliant labeling is the most important factor in consumer acceptance since texture, perceived health, and ethics determine preferences. Clean labeling avoids making misleading health claims and encourages consumer decision-making and product confidence.

### Proposed mechanistic model and insights for advancing G-FN

Texture and functionality of G-FN are controlled by synergistic interactions between hydrocolloids, dietary fibers, and bioactive compounds in starch–protein matrices. Hydrocolloids act as structural mimetics: XG enhances viscosity and dough stability through hydrogen bonding towards starches and proteins to create a shear-thinning gel that enhances water absorption and shelf life and reduces glycemic index. GG enhances cooking quality through hydration shell formation and gluten-like network formation to enhance water-holding capacity and reduce CL. Dietary fibers like inulin and PSY act as modulators through enhanced dough matrix strength, resistant starch, and glycemic control through slow hydrolysis and moisture storage. Excessive inclusion will affect textural integrity, and optimal ratios are essential. Bioactive compounds like polyphenols, lysine, and insect protein act as multifunctional fortifiers through upgrading protein content, elasticity, and thermal stability through starch–protein–polyphenol complexation. Nano- or micro-encapsulation enhances the stability and shelf-life of antioxidants through controlled release and low oxidative degradation. Additionally, determinants of the matrix, such as particle size of the flours employed, amylose: amylopectin ratio, and extent of hydration, significantly affect textural resilience and consumer-perceived quality. Finally, clean-labeling tactics through transparency and regulatory compliance continue to be essential for consumer confidence in the products.

### Innovations and future directions

Advances in G-FN are increasingly being shaped by interdisciplinary research directed toward overcoming the nutritional, structural, and sensory limitations of gluten exclusion. One of the most intriguing strategies is the synergistic use of multifunctional ingredients like HCs, dietary fibers, and bioactive compounds. These hybrid systems, for example, psyllium husk and β-glucan and green tea extract, not only improve dough rheology and cooking quality but also enrich the fiber content and extend antioxidant protection. An in-depth understanding of the physicochemical and rheological interactions between these ingredients is essential to their realization of their full functional potential.

To address the protein deficit in gluten-free matrices, new advances explore the fortification of G-FNs with plant proteins extracted from legumes or other eco-friendly sources. Such proteins enrich the nutritional content and enhance textural characteristics, suitable adjustment with HCs compatibility and thermostability being required for effective incorporation. Also, encapsulation at the nano- and microlevels of bioactive compounds such as polyphenols and omega-3 fatty acids further amplifies the functionality of G-FN by offering antioxidant protection against oxidation degradation, sustained release, and preservation of organoleptic properties throughout processing and storage.

From the sustainability point of view, using agro-industrial waste materials on G-FNs is an interesting way to increase dietary fiber and polyphenol levels and decrease food loss, besides supporting the resource efficiency objectives. Concurrently, tailored G-FN formulations are being created to meet the nutritional requirements of targeted consumer groups. For instance, iron- and calcium-enriched children’s noodles, diabetes-management low-glycemic index foods, and anti-inflammatory-enriched elderly foods. Innovation also includes smart labelling and digital traceability, where technologies like QR-code-based packaging provide consumers with real-time information on ingredient origin, nutritional composition, and allergenicity, facilitating transparency and informed consumption. At the same time, artificial intelligence and computational modelling are transforming the formulation process. These tools enable in silico simulation of ingredient interactions, prediction of product performance, and optimization of nutritional and sensory profiles, thus reducing formulation cost and accelerating time-to-market.

Collectively, these scientific innovations reflect a paradigm change in G-FN and serve as a guide to the development of nutritionally, technologically improved, consumer-relevant, health-conscious, and ecologically compatible G-FN foods. The creation of G-FN relies on the strategic blending of dietary fibers, HCs, and bioactive compounds to replicate the structural and functional properties of gluten. Well-defined formulation strategies, particularly ones derived from underutilised or botanical sources, present a green path toward improving texture, nutritional content, and consumer appeal. Minimally intensive processing technologies that preserve thermolabile nutrients, along with packaging and moisture management developments, continue to improve product stability and shelf life. Regulatory compliance and transparent labelling, which guarantee consumer confidence and safety, are still essential. With the growing global demand for health-oriented G-FN, future research must respond to the enhancement of nutritional quality, sensory properties, and market accessibility, especially in developing nations. By connecting scientific innovation to environmental and regulatory expectations, the G-FN industry stands well-placed to deliver top-quality, functional foods that address both health and environmental needs.

## Supplementary Information

Below is the link to the electronic supplementary material.

Supplementary file1 (DOCX 5896 KB)
