# Supplementary material for: Next-generation gluten-free noodles: integration of hydrocolloids, fibers, and bioactive compounds
Source: Food Sci Biotechnol. 2026 Jan 12;35(8):2097–115. doi: 10.1007/s10068-025-02081-w (PMC13283120; doi:10.1007/s10068-025-02081-w)
Supplement: Supplementary file 1 — Supplementary file1 (DOCX 5896 KB) [file 10068_2025_2081_MOESM1_ESM.docx]

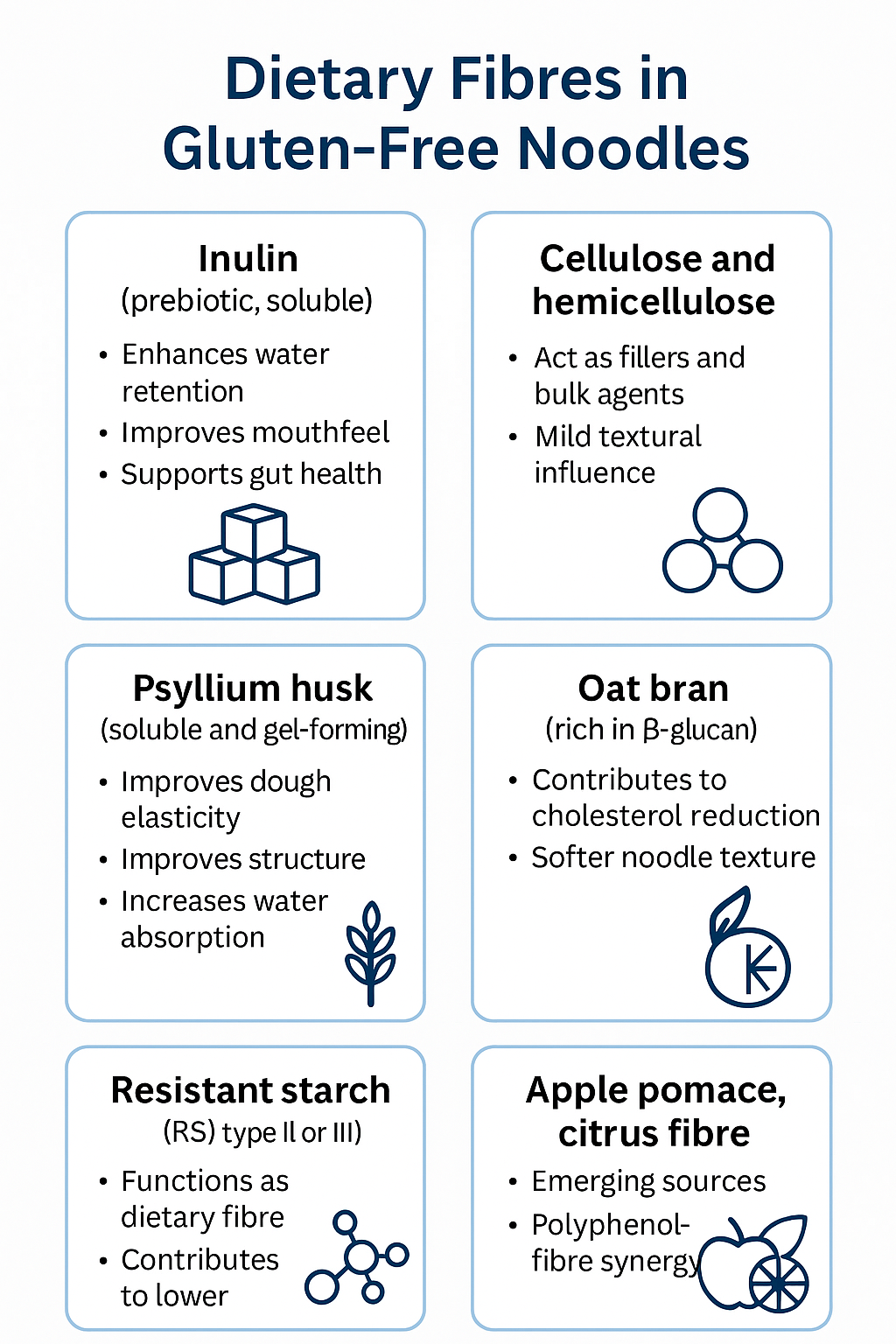


Fig. S1: Dietary fibres in G-FN- advantages and limitations (Adapted from Lazaridou et al., 2007; Ronda et al., 2013; Zhang et al., 2018).


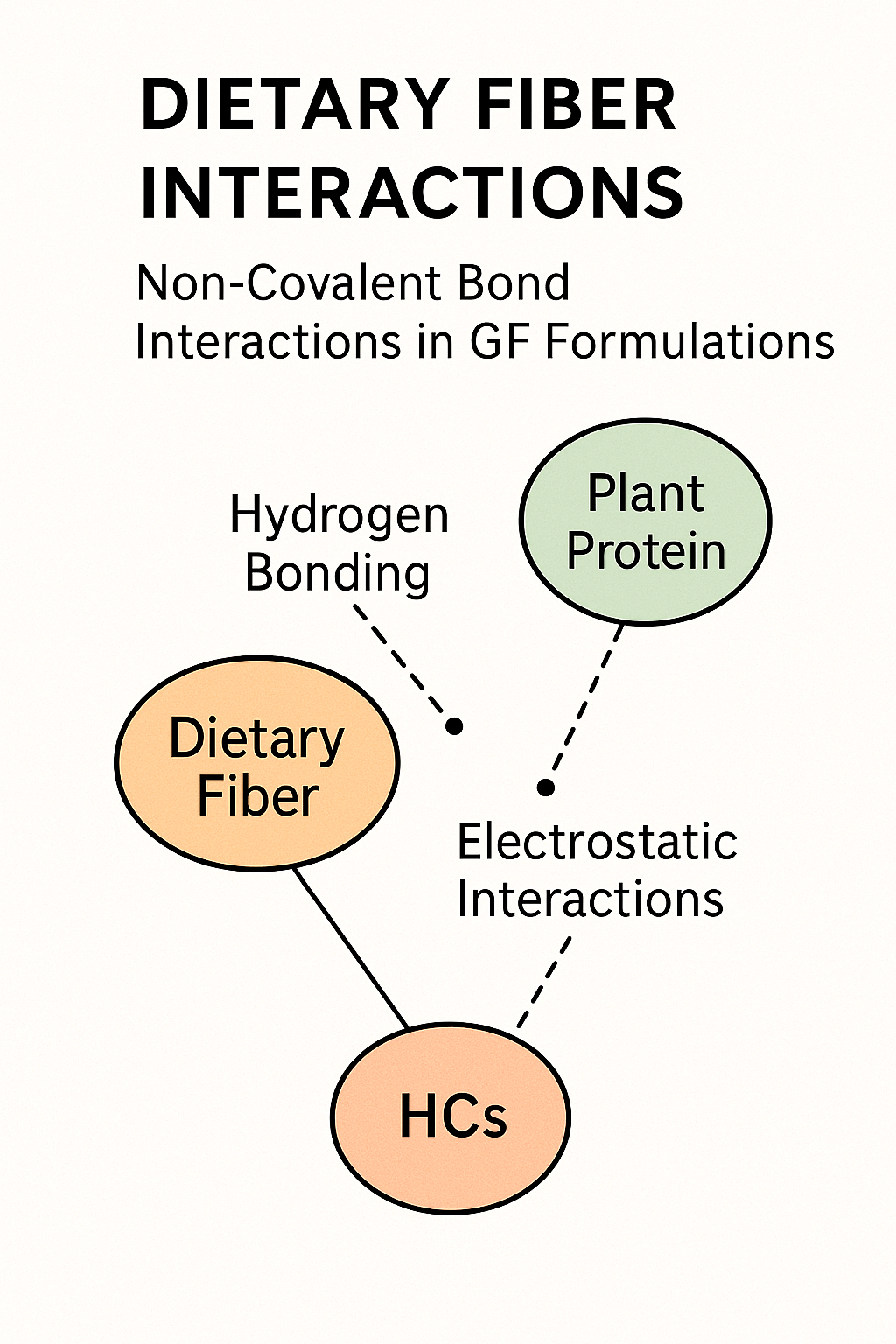


Fig. S2: Dietary fibre interactions in G-FN (non-covalent bond interactions) (modified from Zhang et al., 2018)


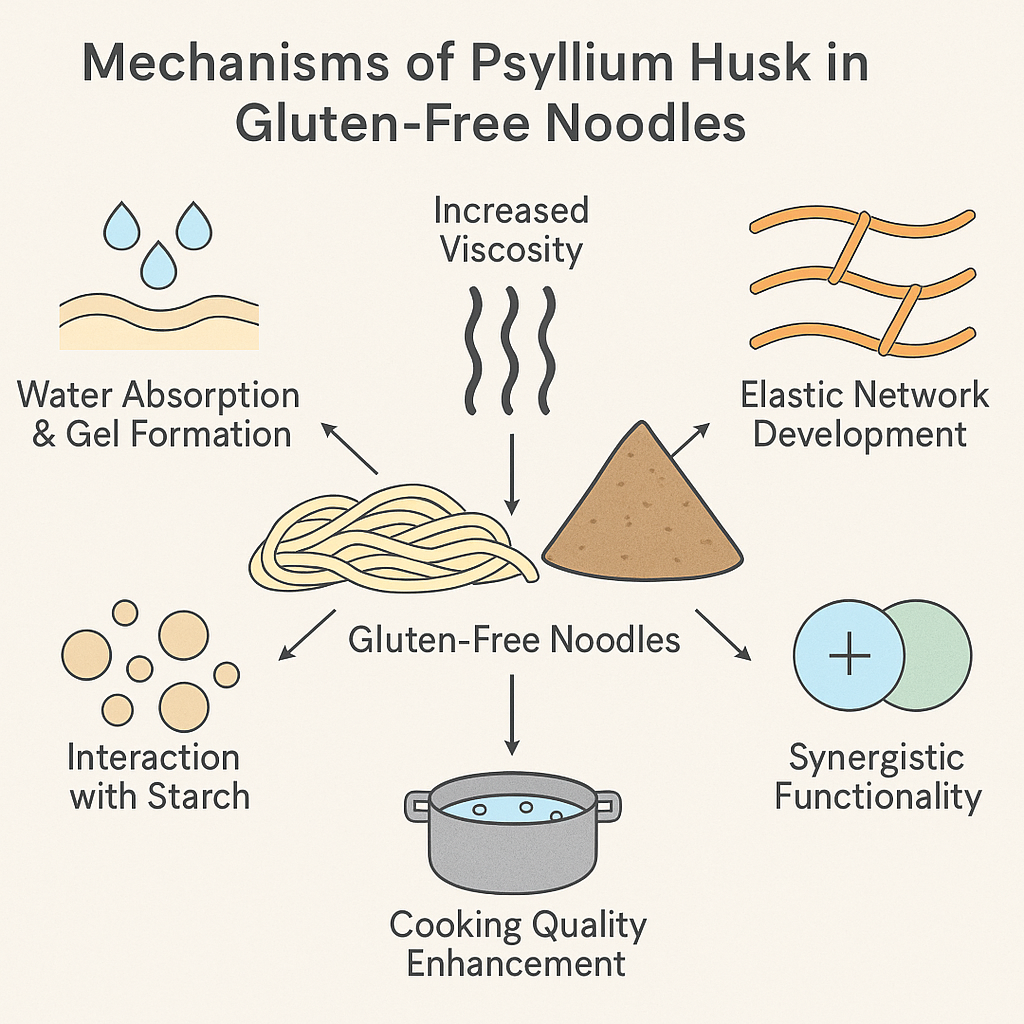


Fig. S3: Roles of fibres (inulin) in G-FN (modified from Chen et al., 2021; Park et al., 2022)


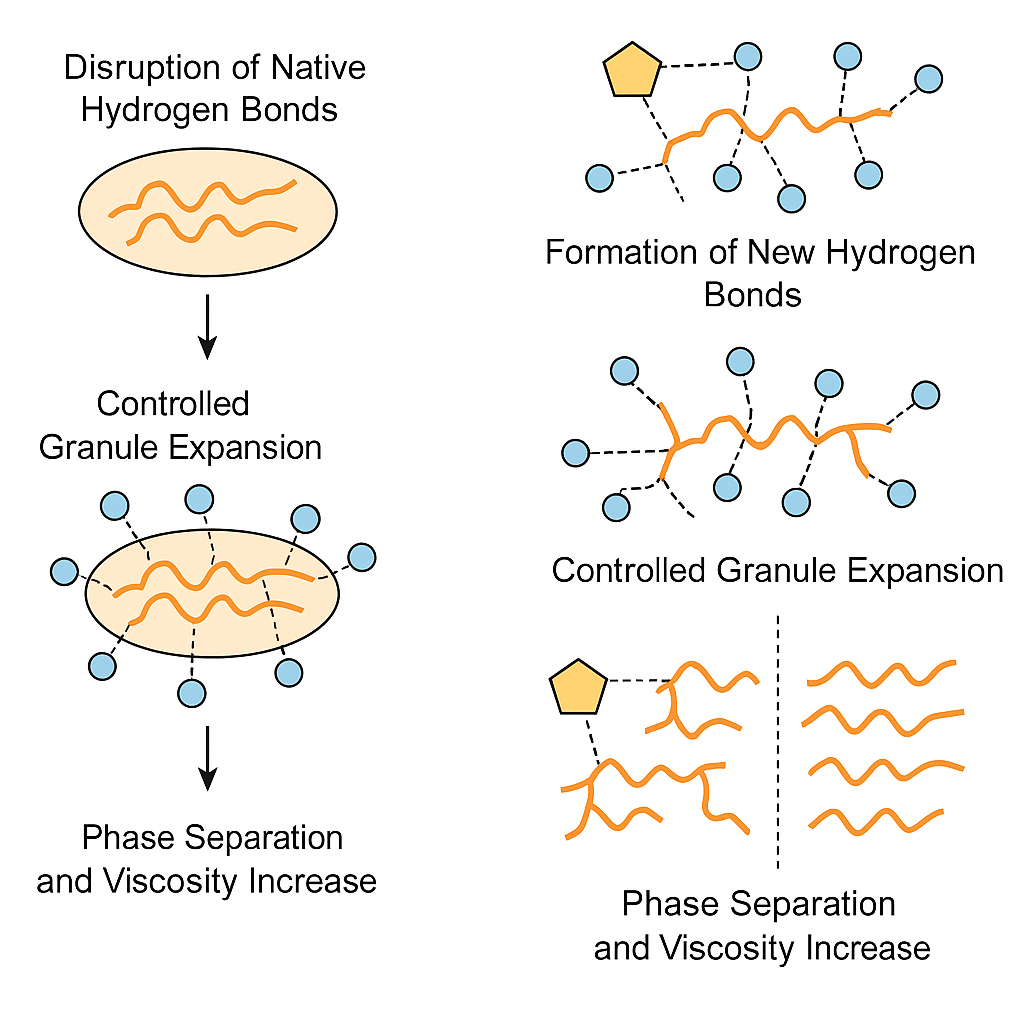


Fig. S4: Mechanisms of interaction of Sugar Alcohols with Starch and Water (modified from Gao et al., 2023; Martinez et al., 2015; Woodbury et al., 2022).
